# The C-terminal dimerization domain of the respiratory mucin MUC5B functions in mucin stability and intracellular packaging before secretion

**DOI:** 10.1074/jbc.RA119.010771

**Published:** 2019-09-30

**Authors:** Caroline Ridley, Michael P. Lockhart-Cairns, Richard F. Collins, Thomas A. Jowitt, Durai B. Subramani, Mehmet Kesimer, Clair Baldock, David J. Thornton

**Affiliations:** ‡ Wellcome Trust Centre for Cell-Matrix Research, The University of Manchester, Oxford Road, Manchester M13 9PT, United Kingdom; § Division of Infection Immunity and Respiratory Medicine, The University of Manchester, Oxford Road, Manchester M13 9PT, United Kingdom; ¶ School of Biological Sciences, Faculty of Biology, Medicine and Health, Manchester Academic Health Science Centre, The University of Manchester, Oxford Road, Manchester M13 9PT, United Kingdom; ‖ Division of Cell-Matrix Biology and Regenerative Medicine, The University of Manchester, Oxford Road, Manchester M13 9PT, United Kingdom; ‡‡ Lydia Becker Institute for Immunology and Inflammation, The University of Manchester, Oxford Road, Manchester M13 9PT, United Kingdom; ** Marsico Lung Institute/Cystic Fibrosis Research Center, University of North Carolina School of Medicine, Chapel Hill, North Carolina 27599-7362

**Keywords:** mucin, mucus, von Willebrand factor, lung, cryo-electron microscopy, small-angle X-ray scattering (SAXS), Mucus obstruction

## Abstract

Mucin 5B (MUC5B) has an essential role in mucociliary clearance that protects the pulmonary airways. Accordingly, knowledge of MUC5B structure and its interactions with itself and other proteins is critical to better understand airway mucus biology and improve the management of lung diseases such as asthma, cystic fibrosis, and chronic obstructive pulmonary disease (COPD). The role of an N-terminal multimerization domain in the supramolecular organization of MUC5B has been previously described, but less is known about its C-terminal dimerization domain. Here, using cryogenic electron microscopy (cryo-EM) and small-angle X-ray scattering (SAXS) analyses of recombinant disulfide-linked dimeric MUC5B dimerization domain we identified an asymmetric, elongated twisted structure, with a double globular base. We found that the dimerization domain is more resistant to disruption than the multimerization domain suggesting the twisted structure of the dimerization domain confers additional stability to MUC5B polymers. Size-exclusion chromatography-multiangle light scattering (SEC-MALS), SPR-based biophysical analyses and microscale thermophoresis of the dimerization domain disclosed no further assembly, but did reveal reversible, calcium-dependent interactions between the dimerization and multimerization domains that were most active at acidic pH, suggesting that these domains have a role in MUC5B intragranular organization. In summary, our results suggest a role for the C-terminal dimerization domain of MUC5B in compaction of mucin chains during granular packaging via interactions with the N-terminal multimerization domain. Our findings further suggest that the less stable multimerization domain provides a potential target for mucin depolymerization to remove mucus plugs in COPD and other lung pathologies.

## Introduction

Polymeric mucins are the major structural component of mucus; a hydrogel that plays a pivotal role in protecting mucosal surfaces. In healthy airways, mucus in conjunction with cilia, protect the airway epithelium from inhaled pathogens, particles, and toxins (1, 2). The polymeric mucins confer the correct rheological properties for the gel to facilitate removal of these inhaled components by mucociliary clearance (MCC).^4^ However, during airway diseases such as asthma, cystic fibrosis (CF), and chronic obstructive pulmonary fibrosis (COPD), mucus concentration is increased which results in an increase in viscoelasticity. The abnormal mucus impairs effective MCC, generating an environment that harbors pathogenic microbes (3–5).

MUC5B and MUC5AC are the major polymeric mucins in respiratory tract mucus (6–9). The focus of this study is MUC5B, which is essential for mucociliary clearance of the lung (10, 11). MUC5B is a large (2–50 MDa), multidomain gel-forming glycoprotein that shares high sequence homology with the cysteine-rich domains of von Willebrand factor (vWF) at its N- and C-terminal regions (12, 13). MUC5B consists of an N-terminal region composed of vWF-like D1-D2-D′-D3 domains, a central proline-threonine-serine-rich mucin domain interspersed with cysteine-rich (Cys) domains, and C-terminal region composed of vWF-like D4-B-C-CK domains (14). The N- and C-terminal regions are important for disulfide bond-mediated polymer assembly.

MUC5B biosynthesis is a multistep process involving CK-domain disulfide-mediated C-terminal dimerization in the endoplasmic reticulum, and extensive *O-*glycosylation of the central mucin domain followed by D3-domain disulfide-mediated N-terminal multimerization in the Golgi apparatus (15–19). The assembled, linear MUC5B polymer is packaged in a compact and cross-linked form inside secretory granules, via noncovalent interactions between N-terminal dimerization domains controlled by calcium ions and acidic pH (19–21). Post-secretion, uncoupling of these mucin-mucin interactions allows transition to an expanded linear chain that is critical for formation of a flowing mucus gel that facilitates lung protection by MCC (1). The supramolecular organization of MUC5B within mucus is not completely described, although recent studies have shown that submucosal gland-derived MUC5B forms assemblies of the linear mucin strands, termed bundles (22).

Compromised MCC is a feature of muco-obstructive disease and there are multiple mechanisms proposed to generate the pathogenic mucus associated with asthma, CF, and COPD; important contributors include concentration of mucin, defective mucin expansion, and covalent cross-linking of the secreted mucin network (20, 23–27). Understanding how airway obstruction can be reversed is crucial to tackling mucus accumulation in the airways. Therefore, elucidating the molecular details of mucin structure, assembly, packaging, and post-secretory expansion is critical in the development of mucolytic therapies that can directly target the mucin polymers that underpin the adherent mucus plugs/plaques. Although the structure and roles of the MUC5B N-terminal multimerization domain in MUC5B assembly and intragranular packaging have been described (19, 21) the roles of other protein-rich regions of MUC5B in airway mucus biology have not been elucidated. Therefore, we have focused on elucidating the structure and functional roles of the C-terminal dimerization domain of MUC5B in polymer stability and intragranular packaging.

Here, using recombinant protein (D4-B-C-CK), we describe the detailed structure of the C-terminal dimerization domain of MUC5B using single particle cryo-electron microscopy (cryo-EM) and small-angle X-ray scattering (SAXS). We demonstrate that the C-terminal dimerization domain of MUC5B has similar structure to that of the related glycoprotein, vWF (28, 29). Moreover, we show that the C-terminal dimerization domain confers extra stability on the MUC5B polymer compared with the N-terminal dimerization domain. Finally, we have gained new insight into the molecular details of MUC5B packaging in secretory granules by using recombinant C-terminal protein to investigate the effect of calcium and pH on (*a*) homotypic interaction between C-terminal dimerization domain and (*b*) heterotypic interaction with recombinant N-terminal multimerization domain.

## Results

### Expression and characterization of MUC5B C-terminal dimerization domain

**Figure 1.**
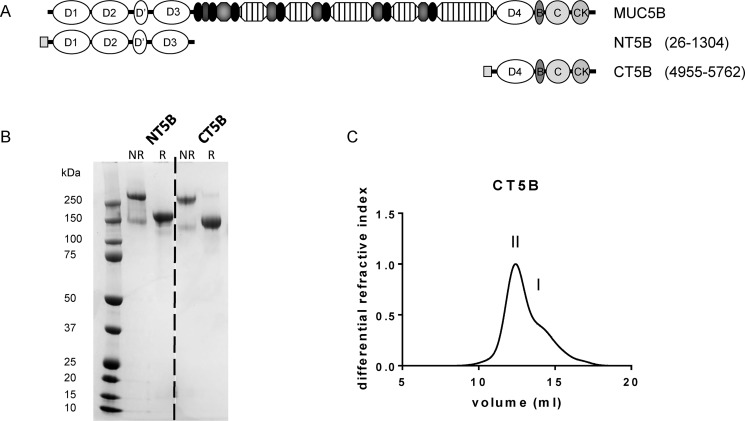
**Expression of recombinant MUC5B protein domains.** *A,* schematic showing the domain structure of N- and C-terminal constructs of MUC5B. The constructs were composed of a N-terminal His_6_ tag (*gray square*) coupled to the MUC5B domains, NT5B (D1-D2-D′-D3) and CT5B (D4-B-C-CK). *B,* N- and C-terminal constructs, reduced (*R*) with 10 mm DTT and nonreduced (*NR*), were analyzed by SDS-PAGE, and stained with InstantBlue. Gel splicing occurred at the position indicated by the *dashed line*, the data came from the same gel. *C,* recombinant CT5B in 0.2 m NaCl, pH 7.4, was analyzed by SEC-MALS and the representative graph shows the differential refractive index. The chromatograph showed two peaks corresponding to dimer (peak *II*) and monomer (peak *I*). Experiments were repeated at least 3 times.

To investigate the structure and further define the role of the C-terminal dimerization domain of MUC5B, we stably expressed a C-terminal construct of MUC5B (CT5B), consisting of D4-B-C-CK domains (Fig. 1*A*). We also expressed the previously published N-terminal dimerization domain of MUC5B (NT5B protein) consisting of D1-D2-D′-D3 domains (19). The expressed proteins were purified from conditioned 293-EBNA medium using a combination of nickel affinity, size exclusion, and anion exchange chromatography. The reduced (R) and nonreduced (NR) proteins were analyzed by SDS-PAGE (Fig. 1*B*) and showed that CT5B was expressed mainly as a disulfide-linked dimer with a small proportion of monomer. The molecular mass for the CT5B dimer (∼245 kDa) and monomer (∼147 kDa) was determined by size exclusion chromatography multi-angle static light scattering (SEC-MALS) (Fig. 1*C*).

### Structural analysis of MUC5B dimerization domain

**Figure 2.**
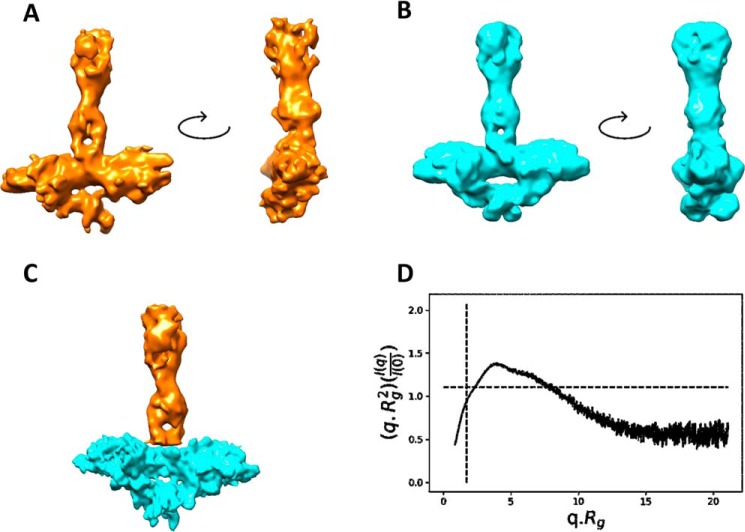
**Structural analysis of MUC5B multimerization domain.** Cryo-EM maps of CT5B refined using either C1 (*A*) or C2 (*B*) symmetry shown with a rotation through 90 degrees. *C,* a composite map of CT5B showing the stalk from the C1 model (*yellow*) and the C2 base (*blue*) from an independent subtracted refinement. *D,* normalized Kratky plot calculated from the SAXS data of CT5B showing a biphasic profile indicating that CT5B has rigid and flexible regions.

**Figure 3.**
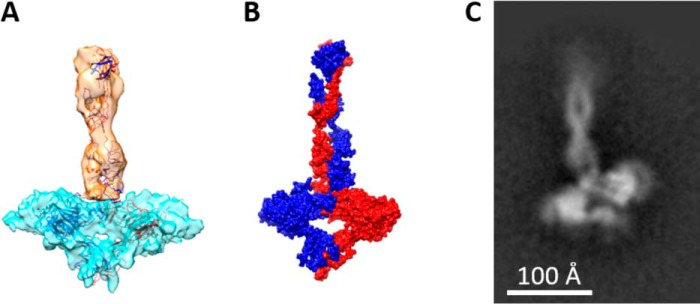
**Structural overview of C-terminal MUC5B.** *A,* the composite map (from Fig. 2*C*) is shown with docked models of each domain. A dimer of the D4 domain fits within the base, with the monomers colored *red* and *blue*. In the stalk, the B-C-CK domains are fitted twisting along the stalk axis, with each monomer colored *orange* and *blue. B*, a surface rendered representation of the docked models shown in *A* with each monomer chain color coded either *red* or *blue. C,* a representative 2D class of CT5B showing this twist along the length of the stalk.

We investigated the structure of dimeric CT5B using cryo-EM and SAXS (Fig. 2 and Figs. S1 and S2). 2D classification of CT5B showed an elongated shape consisting of a globular base connected to an extended stalk region (Fig. 3*C* and Fig. S1*B*). There appeared to be flexibility between the globular base and the stalk as visualized in the 2D classes (Fig. S1*B*). As CT5B is a dimer, the data were processed using either C1 or C2 symmetry. The C1 structure refined to a resolution of 9.3 Å, where the twist in the stalk was clearly visible (Fig. 2*A*), and the C2 structure refined to a resolution of 8.9 Å where the base benefitted from the application of 2-fold symmetry, but the flexible stalk structure deteriorated when symmetry was applied (Fig. 2*B*). Therefore, the stalk was subtracted from the C2 refined structure and locally refined resulting in a gain in resolution to 7.5 Å (Fig. S1*C* and Fig. 3). A composite map of the refined C2 base and the C1 stalk is shown in Fig. 2*C*, where CT5B is a “T” shaped dimeric molecule with dimensions 21 × 18 × 5 nm (height × width × depth).

Analysis of the SAXS data confirmed the flexible nature of CT5B, with the normalized Kratky plot indicating a biphasic system with both globular and elongated regions connected by limited flexibility (Fig. 2*D*). Based on the similarity to the vWF structure and crystal structure of the vWFD domain (28, 29), it was clear that the D4 region was located in the globular base structure. A homology model of the D4 region was therefore generated, which readily docked into the symmetric base (Fig. 3). The D4 domains form a butterfly shape with the termini connecting through the central axis. The CT5B region has 13 predicted *N*-glycans per monomer so some areas where the EM density is not fully occupied by protein structure may reflect the locations of *N*-glycans, which could also add flexibility to the structure (Fig. S3*B*). A resolution of 7.5 Å was achieved despite the heterogeneity contributed by complex *N*-glycosylation and conformational flexibility through the stalk region.

The B-C-CK domains form the stalk structure. It was apparent from the 2D class averages (Fig. 3*C* and Fig. S1*B*) and 3D reconstruction (Fig. 2, *A* and *C*) that the two molecules within the stalk region twist around each other. This may increase the interface and the number of domain-domain interactions between the monomers in the stalk region, potentially increasing the stability of this C-terminal region (Fig. 3). To determine whether the structure of the C-terminal dimerization domain is pH regulated, we analyzed the CT5B dimer by size exclusion chromatography at different pH values (Fig. S4). Results showed no evidence of a change in CT5B structure at different pH values. Taken together, our data suggest that the twist in the stalk of the molecule, which represents the B-C domains, could be a feature that provides enhanced stability to the CT5B dimer.

### Stability of the dimerization and multimerization domains

**Figure 4.**
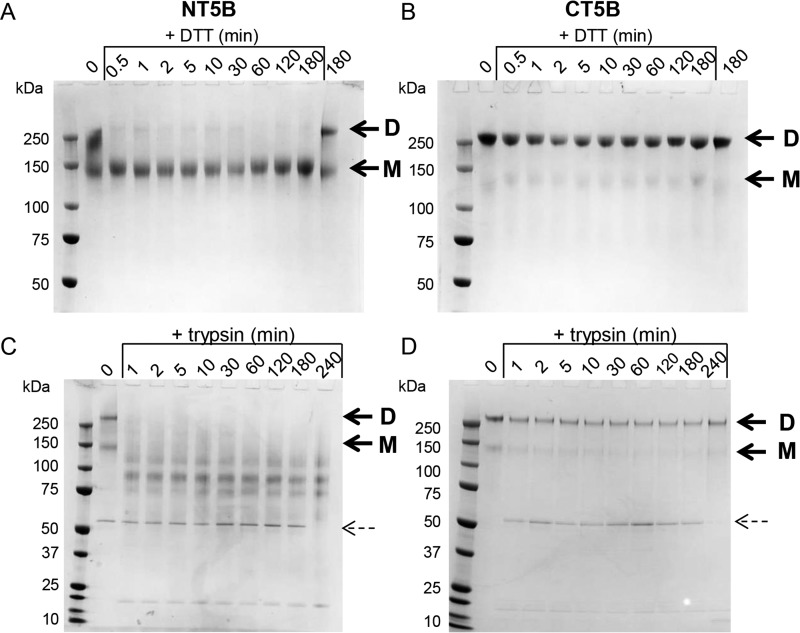
**Stability of the dimerization and multimerization domains of recombinant MUC5B.** *A,* recombinant NT5B and *B,* CT5B were partially reduced by incubation with 1 mm DTT on ice for 0.5 to 180 min. Samples without DTT were incubated for 0 and 180 min. *C,* recombinant NT5B and *D,* CT5B were subjected to limited proteolysis with 1 μg/ml of trypsin for 1 to 240 min at room temperature. Samples without trypsin were incubated for 0 min. The bands visible at approximately 50 and 20 kDa represent the trypsin inhibitor and trypsin, respectively. Dimer (*D*) and monomer (*M*) of NT5B and CT5B are indicated by *arrows*, and trypsin inhibitor is indicated by a *dashed arrow*. The experiments were repeated 3 times.

MUC5B forms linear polymers via disulfide-linked N-N and C-C–terminal interactions. We compared the relative stability of the expressed protein representing the N-terminal multimerization domain of MUC5B (NT5B) (Fig. 1*B*) (19) with CT5B by using limited proteolysis and partial reduction approaches. The NT5B and CT5B proteins were treated with 1 mm DTT on ice (partial reduction; Fig. 4, *A* and *B*) or with 1 μg/ml of trypsin at room temperature (partial proteolysis; Fig. 4, *C* and *D*) and samples collected at different time points were analyzed by SDS-PAGE. Dimer and monomer bands are indicated by *arrows* and trypsin inhibitor (∼50 kDa) is indicated by a *dashed arrow*. Results showed that CT5B dimer was resistant to limited proteolysis and partial reduction, maintaining a dimer throughout the time course of the experiment (Fig. 4, *B* and *D*). In contrast, NT5B dimer was sensitive to both treatments, being reduced to monomer in the presence of DTT after ∼0.5 min (Fig. 4*A*) and lower molecular weight species by the action of trypsin within 1 min (Fig. 4*C*).

**Figure 5.**
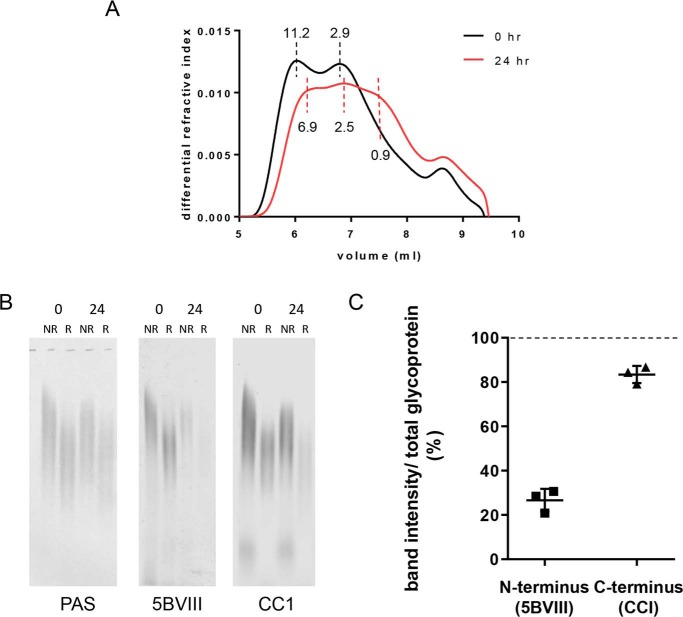
**Stability of the dimerization and multimerization domains of polymeric MUC5B.** MUC5B was incubated with 1 μg/ml of trypsin for 0 and 24 h at 37 °C. *A,* samples were analyzed by SEC-MALS on a Shodex OHpak SB-806 M HQ column. The molecular mass of peaks is indicated (MDa). This experiment was repeated twice. *B,* samples (reduced (*R*) and nonreduced (*NR*)) were analyzed on an agarose gel and transferred to nitrocellulose, then probed with MAN-5BVIII (D3 domain) or CC1 (B domain) antisera or PAS stained. *C,* nonreduced bands were quantified, then MAN-5BVIII and CC1 signals were divided by PAS stain signal (total glycoprotein) and expressed as a percentage compared with the 0 h sample (*dashed line*). *Error bars* represent the S.D. This experiment was repeated 3 times.

To assess whether this differential stability demonstrated with the N- and C-terminal recombinant proteins was observed with native polymers, we analyzed purified MUC5B following proteolysis. SEC-MALS analysis showed that proteolysis caused a reduction in the average molecular mass of MUC5B from 6.8 to 3.5 MDa (Fig. 5*A*). Western blot analysis before and after proteolysis was performed and blots were probed for D3 domain (5BVIII) in the N terminus, and B domain (CC1) in the C terminus, and total glycoprotein was determined by PAS stain (Fig. 5*B*). Results for the unreduced samples showed ∼70% loss of N-terminal antibody signal following proteolysis, whereas the C-terminal antibody signal was only decreased by ∼15% (Fig. 5*C*). A similar trend was observed for the samples that were reduced prior to electrophoresis. These data are consistent with results following trypsin digestion of the recombinant MUC5B proteins (Fig. 4, *C* and *D*) and demonstrate that in native MUC5B polymers the C-terminal dimerization domain is more stable than the N-terminal multimerization domain.

### Effect of calcium and pH on the dimerization domain of MUC5B

We next investigated the potential role of CT5B in MUC5B intragranular packaging. Prior to secretion, linear disulfide-stabilized MUC5B polymers are condensed and packaged within secretory granules that have an acidic pH and high calcium content (1, 19). We previously demonstrated that the disulfide-linked dimeric N-terminal multimerization domains of MUC5B were noncovalently assembled into tetramers through calcium-mediated cross-links, active at acidic pH (pH 5–6) (21). This interaction is proposed to change the organization of the linear polymeric chains and aid their ordered packaging inside secretory granules (19). To determine whether calcium and acidic pH could cause noncovalent interactions between the recombinant C-terminal multimerization domain; dimeric CT5B was incubated in the presence or absence of calcium (5 mm) at pH 7.4 or 6 and analyzed by SEC-MALS (Fig. S5*A*). Results in the presence of EGTA at pH 7.4 and 6 (Fig. 5*A*, *i* and *ii*) showed a peak of dimer (peak II) and monomer (peak I). In the presence of calcium at pH 7.4, 6, or 5, the profiles of CT5B were similar to the data for EGTA and showed that calcium and pH did not facilitate noncovalent interactions between CT5B (Fig. S5*A*, *iii–vi*). These results were also confirmed using analytical ultracentrifugation, which showed monomer (I) and dimer (II) peaks with similar sedimentation profiles for CT5B under all conditions studied (Fig. S5*B*, *i–iii*). These data show that unlike the N-terminal multimerization domain, the C-terminal dimerization domain of MUC5B does not form homotypic calcium or pH-dependent multimers and therefore, does not have the same role as the N-terminal multimerization region in the organized packaging of MUC5B.

### Effect of calcium and pH on the interaction between the dimerization and multimerization domains of MUC5B

**Figure 6.**
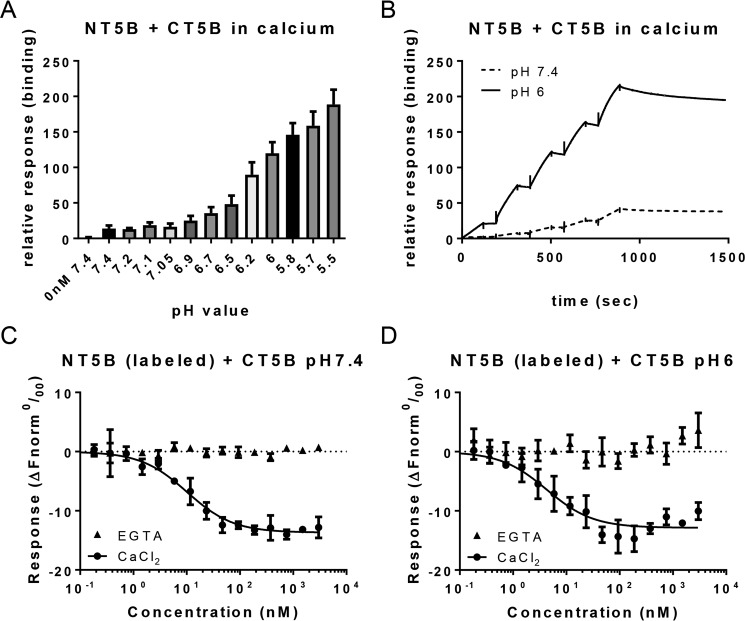
**Analysis of heterotypic interaction of MUC5B dimerization and multimerization domains.** *A,* a pH scan of 50 nm NT5B in different pH buffers in the presence of 5 mm CaCl_2_, flowed over CT5B immobilized to a chip and analyzed using SPR. There was some background binding at pH 7.4, but strong binding occurred between NT5B and CT5B at pH ∼ 6.2. This experiment was performed once with triplicate samples. *B,* single cycle kinetic analysis with increasing concentrations of NT5B in 5 mm CaCl_2_ (0, 5, 10, 15, 20, and 40 nm) at pH 6 (*black line*) or pH 7.4 (*dashed line*). *C* and *D,* serially diluted recombinant CT5B protein was mixed with an equal volume of fluorescently labeled NT5B recombinant protein, and interactions were measured by fluorescence using MST. Samples were prepared in HBS buffer containing 0.05% Tween 20 and either 5 mm CaCl_2_ (*circles*) or 5 mm EGTA (*triangles*) at pH 7.4 (*C*) or 6 (*D*). *Error bars* represent the mean ± S.E. Experiments in *B–D* were repeated at least twice.

Although the presence of calcium and acidic pH did not engender homotypic interaction between CT5B, we hypothesized that the C-terminal multimerization domains of MUC5B may interact with N-terminal dimerization domains during packaging to further facilitate the organization of the mucin polymer inside the secretory granule. Using surface plasmon resonance (SPR), the interaction of immobilized dimeric CT5B with 50 nm dimeric NT5B was analyzed in the presence of 5 mm CaCl_2_ over a range of pH (Fig. 6*A*). This pH scouting assay showed that there was some low background binding between NT5B and CT5B in the presence of calcium at pH 7.4. Binding was shown to increase as a function of decreasing pH, the most binding was at pH ∼ 6.2, and binding was abolished following EDTA treatment. Single cycle kinetic assays were performed with increasing concentrations of NT5B (5-40 nm) flowed over immobilized CT5B in the presence of 5 mm CaCl_2_ at pH 6 and 7.4 (Fig. 6*B*, *solid black line* and *dashed line*, respectively). Analysis showed a strong binding interaction between NT5B and CT5B at pH 6, with a *K_D_* value of ∼1 ± 0.04 nm. At pH 7.4, NT5B flowed over immobilized CT5B with 5 mm CaCl_2_ and produced some binding with a *K_D_* value in a similar range (∼3 nm), however, the binding response was 5-fold lower; suggesting the same binding affinity, but a much lower amount of NT5B bound.

We investigated the heterotypic binding further by performing in solution analysis of the interaction between NT5B (fluorescently labeled) and CT5B (serially diluted) using microscale thermophoresis (MST). The data were analyzed using a *K_D_* model of fit using the NT Affinity Analysis software (Fig. 6, *C* and *D*). The results confirmed that NT5B and CT5B interact in calcium at pH 6, with a *K_D_* of 3 ± 1.4 nm (Fig. 6*D*, *circles*), which was comparable with the result obtained from SPR. The results also showed an interaction at pH 7.4 with a similar binding affinity (*K_D_* 7.4 ± 1.5 nm; Fig. 6*C*). Importantly, there was no interaction detected between NT5B and CT5B in the presence of EGTA at either pH (Fig. 6, *C* and *D*, *triangles*). These results establish that MUC5B dimerization and multimerization domains form reversible pH-sensitive and calcium-dependent intermolecular associations between polymeric mucins.

## Discussion

Determining the nature of polymeric mucin chain interactions during packaging into secretory granules and their subsequent uncoupling post-secretion is critical to understanding the mechanisms that control mucus gel formation in health and disease. Abnormal mucus gel properties may arise due to mucin chains not fully expanding upon secretion, resulting in altered molecular structure. Indeed, airway mucins with aberrant macromolecular structure are a feature of hyper-concentrated mucus that obstructs the CF airways (23). We have previously described a role for the N-terminal multimerization domain of MUC5B in forming noncovalent, reversible calcium-dependent tetramers that are involved in the organization of the mucin chains within the secretory granule (19, 21). Here we have extended understanding of MUC5B intragranular packaging by describing a role for the MUC5B C-terminal dimerization domain.

**Figure 7.**
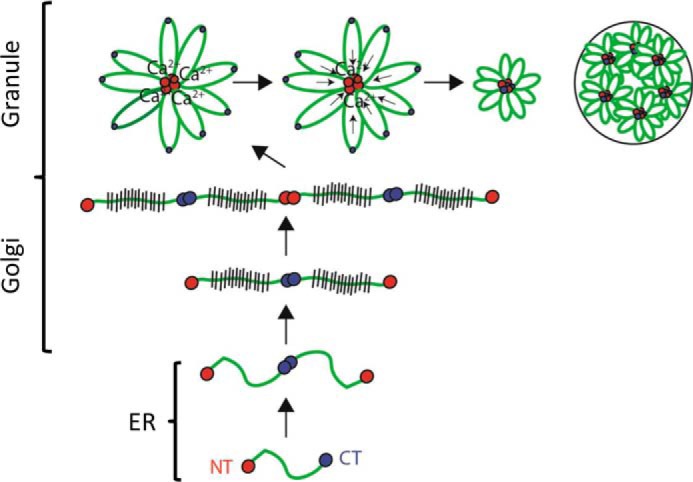
**Model for MUC5B intracellular assembly and packaging.** In the endoplasmic reticulum, MUC5B forms homotypic disulfide-linked dimers via its C-terminal dimerization domains (*blue circles*). The dimer is transported to the Golgi where it undergoes *O-*glycosylation prior to linear polymer formation via disulfide linkage between N-terminal multimerization domains (*red circles*). A reduction in pH and an increase in free calcium concentration occur across the secretory pathway. This results in noncovalent, calcium-mediated interactions between N termini, which appear as proteinaceous nodes, and aid organization of MUC5B for intragranular packaging. The new findings in this paper extend this model and establish that the C-terminal dimerization domains have the potential to interact with the multimerization domains at acidic pH in the presence of calcium, which may aid further compaction of the MUC5B molecule for intragranular packaging. The order of assembly of these N- and C-terminal containing nodes has yet to be elucidated. Uncoupling of these noncovalent interactions in mucus is critical for the transition to an expanded linear chains underpinning normal flowing mucus that can be transported by MCC to keep the lung free from infection. Addition of agents to mucus that displace calcium from mucins has been shown to normalize mucin conformation and decrease mucus viscoelasticity and improve MCC (64, 65).

During packaging of MUC5B within secretory granules, the mucin chains are organized around protein nodes formed from pH and calcium-dependent, noncovalent, reversible, homotypic interactions between N-terminal multimerization domains (19, 21). In marked contrast, we demonstrate that the C-terminal dimerization domain of MUC5B does not form homotypic multimers in the presence of calcium at acidic pH, suggesting it has a different function in intragranular packaging. Indeed, using SPR and MST we demonstrated the potential for reversible, calcium-dependent heterotypic interactions between the dimerization and multimerization domains of MUC5B most active at acidic pH. We propose that these heterotypic interactions aid further compaction of MUC5B during packaging, adding an additional level of organization of the mucin polymers inside the secretory granule (Fig. 7). Further work is required to investigate whether there are any other protein-protein interactions, perhaps with the internal Cys domains of MUC5B (7 in total), that contribute to the full mechanism of packaging and subsequent unfurling of mucins into mucus.

Although the structure of the N-terminal multimerization domain of MUC5B has been described (19, 21), there is little information on the C-terminal dimerization domain. To address the gap in our knowledge, the structure of the C-terminal dimerization domain of MUC5B was determined using SAXS and cryo-EM analysis, and was identified as an anisotropic, extended structure with globular domains. The stalk section of the structure contained a twist, which we interpret as a key structural feature that is likely to contribute to stabilization of the dimerization domain. The related C-terminal region of vWF has a similar shape, described as an extended dimeric stem with bouquet structure (28). However, there are certain differences between MUC5B and vWF C-terminal structures. In vWF, there is no evidence of a twist in the stem-like structure, although interactions have been hypothesized between each pair of tandem C domains (30). Also, at neutral pH encountered during secretion, the vWF stem-like structure unzips, remaining disulfide-bonded at the CK domain (28). Our results showed that the C-terminal structure of MUC5B was not subject to pH-dependent changes, and maintained a stable conformation, potentially aided by the noncovalent bonded, twisted structure of the C domain. These differences between the two molecules may be attributed to the presence of a larger number of B and C domains in the C terminus of vWF (D4-B-B-B-C-C-CK) compared with MUC5B (30–33) and the distinct roles of the two molecules. The resolution of our cryo-EM data are most likely limited by the flexibility of the protein, as shown in the 2D classes (Fig. S1*B*) and by the normalized Kratky plot (Fig. 2*D*), and the large proportion of heterogeneity as a result of glycosylation across the molecule (18).

This study is the first to describe the structure of the MUC5B mucin dimerization domain and it may form a model for other members of the polymeric mucin family. The C-terminal of MUC5B shares some sequence homology with the other polymeric mucins MUC2 and MUC5AC (34–37). However, MUC2 and MUC5AC contain a GDPH autocatalytic cleavage sequence in their D4 domain that is absent in MUC5B (the equivalent sequence is GGSH). The GDPH site is cleaved at acidic pH for MUC2, and neutral to acidic pH for MUC5AC, producing reactive ends that may be involved in forming self-interactions, interactions with other molecules and/or the epithelial cell surface (38, 39). There is no evidence in the literature for C-terminal cleavage in MUC5B. Therefore, there are potential differences in the stability of the C-terminal dimerization domains of MUC2 and MUC5AC compared with MUC5B, which may affect their function.

On comparing the stability of the N- and C-terminal regions of MUC5B, results showed that the C-terminal dimerization domain was more resistant to degradation than the N-terminal multimerization domain, this was seen with both recombinant proteins and the native MUC5B polymer. We have shown here that the central stalk of the dimerization domain has a twisted structure, whereas the multimerization domain has a more open conformation in a boomerang-like shape (19). Thus, the differences in structure correlate well with the differences noted in their stability. These findings provide new insight into the structure and stability of the MUC5B polymer, which may benefit future therapeutic development to alleviate pathology associated with airway mucus obstruction. Indeed, there are already reports of mucolytic agents that directly target the integrity of airway mucin polymers to improve airway clearance by reducing the viscosity of abnormal mucus (40–42). The determination of the differential structure and stability of the critical regions involved in MUC5B polymer assembly offers potential to design more targeted mucoactive agents. In this case, the multimerization domains of MUC5B appear to provide a better target, than the more stable dimerization domains, for production of a mucolytic agent to breakdown mucin polymers and thereby reduce mucus accumulation and improve lung function.

In summary, this study has elucidated a potentially new role for the C-terminal dimerization domain of MUC5B in aiding compaction of mucin chains during granular packaging, via heterotypic interactions with the N-terminal multimerization domains. Results have determined a detailed structure for the dimerization domain of MUC5B, which has shown some similarity with the related vWF C-terminal structure. The structural data have highlighted a central twist in the structure of the dimerization domain, and we propose that this feature contributes to enhanced stability of this region of the MUC5B polymer.

## Experimental procedures

### Expression and purification of recombinant human MUC5B protein domains

MUC5B (UniProtKB accession number Q9HC84) C-terminal construct consisting of D4-B-C-CK (CT5B; residues 4955–5762) (12) was created with an N-terminal His_6_ tag using the mammalian episomal expression vector pCEP-His in 293-EBNA cells (19, 43, 44) (Fig. 1*A*). The N-terminal construct (NT5B consisting of D1-D2-D′-D3) has been described previously (19) (Fig. 1*A*). A Superose 6 10/300 GL column was used for size fractionation. Protein identity and purity were confirmed by tandem MS. Samples, with and without treatment with 10 mm for 4 min at 95 °C, were analyzed by SDS-PAGE using 4–12% BisTris gels (Life Technologies) and stained with InstantBlue (Expedeon).

### SEC-MALS

For determination of molecular mass by SEC-MALS analysis, recombinant proteins were applied to a Superose 6 10/300 GL column and MUC5B was applied to a Shodex OHpak SB-806 M HQ column, in 0.2 m NaCl, 0.05 m EDTA, 0.05% sodium azide. For experiments in the presence of calcium and EGTA, recombinant CT5B protein was incubated overnight at 4 °C in 5 mm CaCl_2_ or 5 mm EGTA at pH 7.4 or 6 in 25 mm HEPES, 150 mm NaCl, before applying to a Superose 6 10/300 GL column at room temperature. For samples at pH 6 and 5, the HEPES was replaced with 25 mm MES. Column eluents passed through an in-line Helios 18-angle laser photometer and a T-rEX refractometer with QELS dynamic light scattering attachment (Wyatt Technology). Analysis was performed using ASTRA version 6 software.

### Analytical ultracentrifugation

Recombinant CT5B protein was incubated in the presence of 5 mm CaCl_2_ overnight at 4 °C in either HEPES or MES buffer (25 mm HEPES, 150 mm NaCl, pH 7.4, or 25 mm MES, 150 mm NaCl, pH 6 or pH 5). Samples were analyzed using velocity experiments on an Optima XL-A ultracentrifuge (Beckman Instruments), as previously described (19). The sedimentation coefficients were determined using SedFit version 13.0b (45).

### Cryo-EM

#### 

##### Grid prep, regular grids

Quantifoil R1.2/1.3 holey carbon grids were cleaned in 2× chloroform soaks on filter paper in a glass Petri dish and then glow discharged for 2 min at 25 mA. 3 μl of purified CT5B at 0.25 mg/ml was applied to the grids and blotted for 8–10 s at 22 °C in a Vitrobot Mark IV (Thermo Fisher Scientific), before plunge freezing in liquid ethane.

##### Data acquisition

Cryo-EM data were collected on a Titan Krios electron microscope operating at 300 kV equipped with a K2 Summit direct detector (Gatan) and a FEI Volta phase plate (Thermo Fisher Scientific) at the Electron Bio-Imaging Centre (eBIC) (Didcot, UK). EPU (Thermo Fisher Scientific) was used to automate the collection of 1441 movies, comprising of 40 frames, 10 s exposures, and a total dose of 40 *e*^−^ Å^2^ were recorded on the detector in counting mode at a calibrated magnification of 93,000 corresponding to a magnified pixel size of 1.043 Å. Defocus was maintained at −0.5 μm as suggested previously (46).

##### Data processing

The recorded movies were processed in cryoSPARC v2.8 (47, 48). Movies were aligned using local patch-based motion correction. CTF estimation of the aligned movie stacks was carried out using the local patch-based CTF estimation. Images were then excluded based on motion distance, resolution fit, defocus, and phase shift resulting in 1093 images. Several hand-picked particles were submitted to 2D classification and used for template-based picking over the entire data set. 231,589 particles were extracted after per particle motion correction in a 500 pixel box. Particles were submitted to two rounds of 2D classification and resulted in 16,590 particles. These particles were used to generate an *ab initio* model for further refinement. Refinement was carried out using C1 and C2 symmetries resulting in structures with resolutions of 10.0 and 9.4 Å, respectively. Heterogeneous refinement was then applied to each result with modest differences between the classes. The particles from the best classes were then used to re-refine the structure resulting in lower resolution structures. Non-uniform refinement was then used to refine the C1 and C2 refined models resulting in a resolution of 9.3 and 8.9 Å, respectively. Particle subtraction was then employed to enhance the resolution of each of the base or stalk regions using masks around the base and stalk. Further refinement of both subtracted symmetry models were completed yielding a base with a resolution of 7.5 Å. The stalk, in the absence of the base, was refined but resulted in a much lower resolution map.

### Model generation

The sequence of CT5B (residues 4955–5762) was submitted to the HHpred server (49–51) through the new MPI Bioinformatics Toolkit online portal (52). HHpred was able to identify structures in the PBD (53) with homology equating to 97% of the CT5B sequence (784 residues of 807). The homologous structures showed high probability with values greater that 95%. The corresponding PDBs (Table 1) and sequence were then submitted to Modeller (54) though the online portal. The resulting models were downloaded and manually placed into the cryo-EM density map using Chimera (55). As the C-CK domains contain an intramolecular disulfide bond in the cysteine knot, a homology model of the C-CK domain was generated in SWISS-MODEL (56), which generated a homology model based on chain B of the second biological assembly of PDB 5BPU (57); the C-CK homology model was produced with an intramolecular disulfide bond. Glycans were identified on the full sequence using NetNGlyc server (www.cbs.dtu.dk/services/NetNGlyc/).^5^

**Table 1:** **Modeling the domains of CT5B based on structural homology** The table shows the percentage identity between domains in CT5B and the corresponding structures from the PDB.

Target length	Probability	PDB code (chain)	Citation
	%		
483	100	6N29 (B)	60
103	95.3	2MHQ (A)	61
85	98.3	6FWN (A)	62
113	98.8	2KD3 (A)	63

### SAXS

SAXS intensity data, *I(q*) *versus q* (*q* = 4π. *sin* 2θλ), of CT5B were collected using SEC-SAXS on beamline B21 (Diamond Light Source, UK). 50 μl of BMPER was loaded onto a Superdex 200 Increase 3.2/300 column (GE Healthcare) at 0.075 ml/min. SAXS data were collected at 3-s intervals on an Eiger X 4M detector (Dectris) at a distance of 2.7 m and wavelength of 0.95 Å.

Data were reduced using in-house software. Subtractions of the SEC-SAXS data were completed for each frame across the elution peak and the radius of gyration (*R_g_*) and the integral of intensity ratio to background were plotted. The data were scaled, merged, and averaged for each frame with a consistently similar *R_g_*. All further processing and analysis of data were carried out using ScÅtter (www.bioisis.net/scatter)^5^ (66).

### Partial reduction and limited proteolysis of recombinant MUC5B proteins

For partial reduction experiments, NT5B and CT5B proteins were incubated with 1 mm DTT on ice between 0.5 and 180 min. At each time point, 2.5 mm iodoacetamide was added and incubated for 15 min in the dark at room temperature. For limited proteolysis, NT5B and CT5B proteins were incubated with 1 μg of sequencing-grade trypsin (Promega) at room temperature for 1 to 240 min. At each time point, 1 μl of trypsin neutralizing solution (Lonza) was added to stop the reaction. Samples were analyzed by SDS-PAGE using 4–12% BisTris gels (Life Technologies), staining with InstantBlue (Expedeon).

### Partial proteolysis of native MUC5B

MUC5B was purified from A549MUC5Bm (A549 cell line in which MUC5AC was knocked down by gene editing) cell-conditioned medium using cesium chloride isopycnic centrifugation in 0.1 m NaCl, as previously described (58). The MUC5B was further purified on a Sepharose CL2B size exclusion column. Partial proteolysis of purified MUC5B was achieved by incubation with 1 μg of trypsin at 37 °C for 0 and 24 h. Trypsin neutralizing solution (1 μl; Lonza) was added to samples to stop the reaction. Samples (reduced (R) and nonreduced (NR)) were equally loaded in triplicate and analyzed on 0.7% (w/v) agarose gels in 40 mm Tris acetate, 1 mm EDTA, pH 8.0, containing 0.1% SDS, for 16 h. Following electrophoresis, gels with unreduced samples were incubated with 10 mm DTT in 4× SSC, pH 8.0, for 15 min. MUC5B was transferred to nitrocellulose membrane by vacuum blotting at 45 millibars for 1.5 h. The membrane was cut into strips and either probed with polyclonal MAN-5BVIII antiserum (19), which detects the N-terminal D3 domain and CC1 antiserum, which detects C-terminal B domain, at 1:2000 dilution. A secondary IRDye® 800CW antibody (LI-COR Biosciences) was used for detection and blots were analyzed on the LI-COR Odyssey® CLx and quantified using Image Studio Lite v5.2 software (LI-COR Biosciences). The final strip was stained using PAS as previously described (59), and bands were imaged on a Bio-Rad ChemiDoc^TM^ MP and quantified using Image Lab v5.2 software (Bio-Rad Laboratories). The nonreduced bands were quantified, and a ratio of the signal was determined for 5BVIII and CC1 relative to PAS signal.

### SPR

To investigate interactions between NT5B and CT5B proteins, a Biacore T200 biosensor with Biacore Control software was used (GE Healthcare). Initially 25 μg/ml of CT5B diluted in 50 mm sodium acetate, pH 5.5, was immobilized onto a CM5 sensor chip by amine coupling using 1-ethyl-3(3-dimethylaminopropyl)-carbodiimide hydrochloride, *N*-hydroxysuccinimide, and ethanolamine-HCl (GE Healthcare). The immobilized CT5B gave ∼3400 response units. To perform a pH scan experiment, purified NT5B protein (50 nm) was injected over the immobilized CT5B in varying pH buffers containing 5 mm CaCl_2_ and 0.05% Tween 20 for 120 s at a flow rate of 30 μl/min and dissociation time of 60 s. The CM5 sensor chip was regenerated after each sample application with 10 mm EDTA, pH 7.4, at a flow rate of 30 μl/min for 30 s and stabilization of 1 min after regeneration. For single cycle kinetic analysis, 0, 5, 10, 15, 20, and 40 nm NT5B analyte was flowed over the immobilized CT5B in HBS containing 5 mm CaCl_2_ and 0.05% Tween 20, at pH 6 or 7.4. Curves were fitted using 1:1 Langmuir association/dissociation model (Biacore Evaluation 4.1 software; GE Healthcare) to obtain kinetic values of binding affinity.

### MST

Purified NT5B protein in 25 mm HEPES, 150 mm NaCl, pH 7.4, was fluorescently labeled using the Monolith^TM^ NT protein labeling kit RED-NHS (Amine Reactive) dye, following the manufacturers' instructions (NanoTemper Technologies). All stock protein samples were centrifuged for 10 min prior to setting up the experiment. The labeled NT5B protein was diluted to 2.5 nm final concentration, in the appropriate buffer for each experiment (25 mm HEPES, 150 mm NaCl, 0.05% Tween 20, with either 5 mm CaCl_2_ or 5 mm EGTA, at pH 7.4 or 6). The nonlabeled recombinant CT5B protein was serially diluted 1:1 in the same buffer (starting concentration of 6 μm) and an equal volume of the labeled protein was mixed with each dilution. The samples were loaded onto Monolith^TM^ NT.115 Standard Treated Capillaries (NanoTemper Technologies) and thermophoresis was measured using a Monolith^TM^ NT.115^PICO^ instrument (NanoTemper Technologies) with NT Control software version 1.0.1 at room temperature with 5 s/30 s/5 s laser off/on/off times, respectively, and 15% LED power and 40% IR-laser (MST) power. Experiments were carried out in triplicate and data from three independently pipetted measurements were analyzed using NT Affinity Analysis software version 2.0.2, using the *K_D_* model of fit.

## Author contributions

C. R., M. P. L.-C., R. F. C., and T. A. J. data curation; C. R., M. P. L.-C., R. F. C., T. A. J., and C. B. formal analysis; C. R., M. P. L.-C., R. F. C., and T. A. J. validation; C. R., M. P. L.-C., R. F. C., and T. A. J. investigation; C. R., M. P. L.-C., and R. F. C. visualization; C. R. and D. J. T. writing-original draft; C. R. and D. J. T. project administration; C. R., M. P. L.-C., R. F. C., T. A. J., C. B., and D. J. T. writing-review and editing; D. B. S. and M. K. resources; C. B. and D. J. T. supervision; D. J. T. conceptualization; D. J. T. funding acquisition.

## Supplementary Material

Supporting Information

## Footnotes

5 Please note that the JBC is not responsible for the long-term archiving and maintenance of this site or any other third party hosted site.

## Footnotes

4 The abbreviations used are:

MCC: mucociliary clearance
CF: cystic fibrosis
COPD: chronic obstructive pulmonary disease
vWF: von Willebrand factor
SAXS: small angle X-ray scattering
SEC-MALS: size exclusion chromatography multi-angle static light scattering
PAS: periodic acid-Schiff
SPR: surface plasmon resonance
MST: microscale thermophoresis
PDB: Protein Data Bank
BisTris: 2-[bis(2-hydroxyethyl)amino]-2-(hydroxymethyl)propane-1,3-diol.
