# Supplementary material for: The C-terminal dimerization domain of the respiratory mucin MUC5B functions in mucin stability and intracellular packaging before secretion
Source: J Biol Chem. 2019 Sep 30;294(45):17105–16. doi: 10.1074/jbc.RA119.010771 (PMC6851316; doi:10.1074/jbc.RA119.010771)
Supplement: Supporting Information [file supp_294_45_17105__index.html]

The C-terminal dimerization domain of the respiratory mucin MUC5B functions in mucin stability and intracellular packaging before secretion — MUC5B structure, stability and interactions — The C-terminal dimerization domain of the respiratory mucin MUC5B functions in mucin stability and intracellular packaging before secretion — MUC5B structure, stability, and interactions — Supporting Information 

# The C-terminal dimerization domain of the respiratory mucin MUC5B functions in mucin stability and intracellular packaging before secretion

## Supporting Information

- EM Data bank validation report - Full wwPDB/EMDataBank EM Map Validation Report
- Supporting Information (to be published online) - Supporting figures
