# Supplementary material for: The C-terminal dimerization domain of the respiratory mucin MUC5B functions in mucin stability and intracellular packaging before secretion
Source: J Biol Chem. 2019 Sep 30;294(45):17105–16. doi: 10.1074/jbc.RA119.010771 (PMC6851316; doi:10.1074/jbc.RA119.010771)
Supplement: Supporting Information [file supp_RA119.010771_155406_1_supp_398201_py3c5p.pdf]

Full wwPDB/EMDataBank EM Map Validation Report (i)

Aug 28, 2019 – 12:45 pm BST

EMDB ID:	:	EMD-10264
Title	:	Respiratory mucin MUC5B; C-terminal dimerization domain structure
Deposited on	:	2019-08-28
Resolution	:	10.00 Å(reported)

This is a Full wwPDB/EMDataBank EM Map Validation Report.

This report is produced by the wwPDB biocuration pipeline after annotation of the structure.

We welcome your comments at *validation@mail.wwpdb.org*
A user guide is available at
https://www.wwpdb.org/validation//EmDataBankMapValidationReportHelp
with specific help available everywhere you see the (i) symbol.

CONFIDENTIAL VALIDATION REPORT

1 Experimental information (i)

Property	Value	Source
Reconstruction method	SINGLE PARTICLE	Depositor
Imposed symmetry	POINT, C1	Depositor
Number of particles used	16590	Depositor
Resolution determination method	FSC 0.143 CUT-OFF	Depositor
CTF correction method	NONE	Depositor
Microscope	FEI TITAN KRIOS	Depositor
Voltage (kV)	300	Depositor
Electron dose ($e^-/\text{Å}^2$)	40	Depositor
Minimum defocus (nm)	Not provided	Depositor
Maximum defocus (nm)	Not provided	Depositor
Magnification	Not provided	Depositor
Image detector	GATAN K2 SUMMIT (4k x 4k)	Depositor

CONFIDENTIAL VALIDATION REPORT
