# Supplementary material for: The C-terminal dimerization domain of the respiratory mucin MUC5B functions in mucin stability and intracellular packaging before secretion
Source: J Biol Chem. 2019 Sep 30;294(45):17105–16. doi: 10.1074/jbc.RA119.010771 (PMC6851316; doi:10.1074/jbc.RA119.010771)
Supplement: Supporting Information [file supp_RA119.010771_155406_1_supp_401563_pyfylf.pdf]

SUPPLEMENTARY MATERIAL

The C-terminal dimerization domain of the respiratory mucin MUC5B functions in mucin stability and intracellular packaging before secretion

***Caroline Ridley[1,3,5], *Michael P Lockhart-Cairns[1,4,5], Richard F Collins[5], Thomas A Jowitt[1,5], Durai B Subramani[6], Mehmet Kesimer[6], Clair Baldock[1,4,5#], and David J Thornton[1,2,3,5#]**

*Joint first authors

[1]Wellcome Trust Centre for Cell-Matrix Research, [2]Lydia Becker Institute for Immunology and Inflammation, [3]Division of Infection Immunity and Respiratory Medicine, [4]Division of Cell-Matrix Biology and Regenerative Medicine, [5]School of Biological Sciences, Faculty of Biology, Medicine and Health, Manchester Academic Health Science Centre, The University of Manchester, Oxford Road, Manchester, M13 9PT, [6]Marsico Lung Institute/Cystic Fibrosis Research Center, University of North Carolina School of Medicine, Chapel Hill, NC, USA, 27599-7362.

Running title: *MUC5B structure, stability and interactions*

[#]To whom correspondence: should be addressed:
David J Thornton, Division of Infection Immunity and Respiratory Medicine, School of Biological Sciences, Faculty of Biology, Medicine and Health, The University of Manchester, Oxford Road, Manchester, M13 9PT; dave.thornton@manchester.ac.uk; Tel. +44 (0)161 275 5647.
Clair Baldock, Division of Cell-Matrix Biology and Regenerative Medicine, School of Biological Sciences, Faculty of Biology, Medicine and Health, The University of Manchester, Oxford Road, Manchester, M13 9PT; clair.baldock@manchester.ac.uk; Tel. +44 (0)161 275 5439

Keywords: Mucin, Mucus, Lung, Mucus obstruction, von Willebrand factor (vWF), mucociliary clearance, obstructive lung disease, hydrogel, secretory granule, cystic fibrosis

---

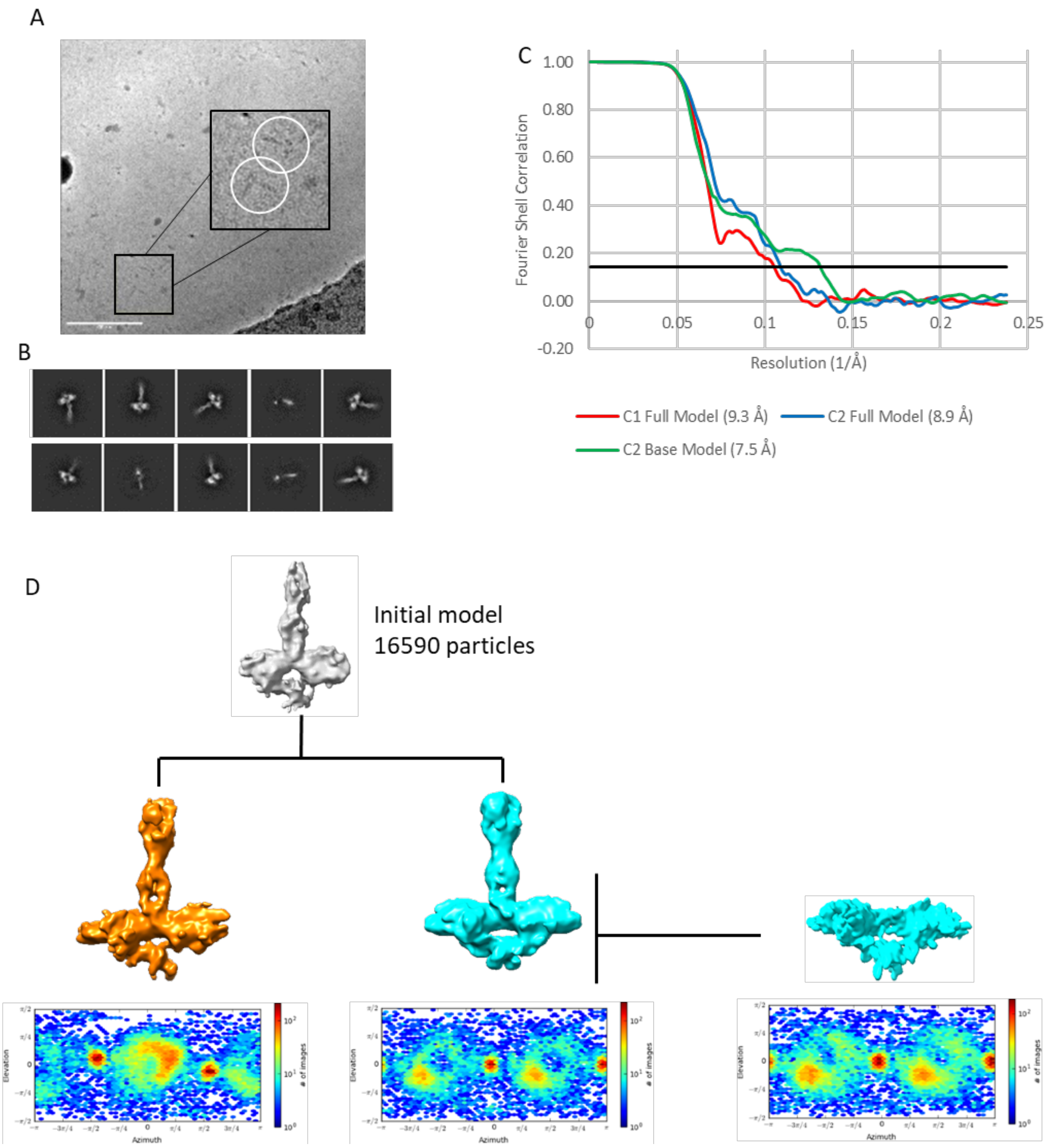

Supplementary Figure 1: Schematic of CT5B single particle cryo-EM data processing
(A) A typical micrograph with an inset showing particle representations. Scale bar = 100 nm. (B) Representative reference-free 2D class averages, in 52 nm x 52 nm boxes, showing clearly the twist in the stalk of the protein and flexibility. (C) Fourier shell correlation of CT5B refined with C1 and C2 symmetries, and a local refine of a C2 symmetry base after particle subtraction. Resolution was calculated from the correlation between two independently refined halves of the data (gold-standard FSC). (D) Around 185000 particles were auto-picked from templates resulting from manually picked particles. Final classes containing 16590 particles were selected for further refinement. Refinement of the initial model was completed with a full mask, and with a mask around the base. Refinement of the full volume of CT5B resulted in a resolution

of 9.87 Å with some anisotropy due to particle orientation bias. The base of CT5B was refined to 7.5 Å and showed similar isotropy. The respective angular distribution of particles is shown below each of the reconstructions.

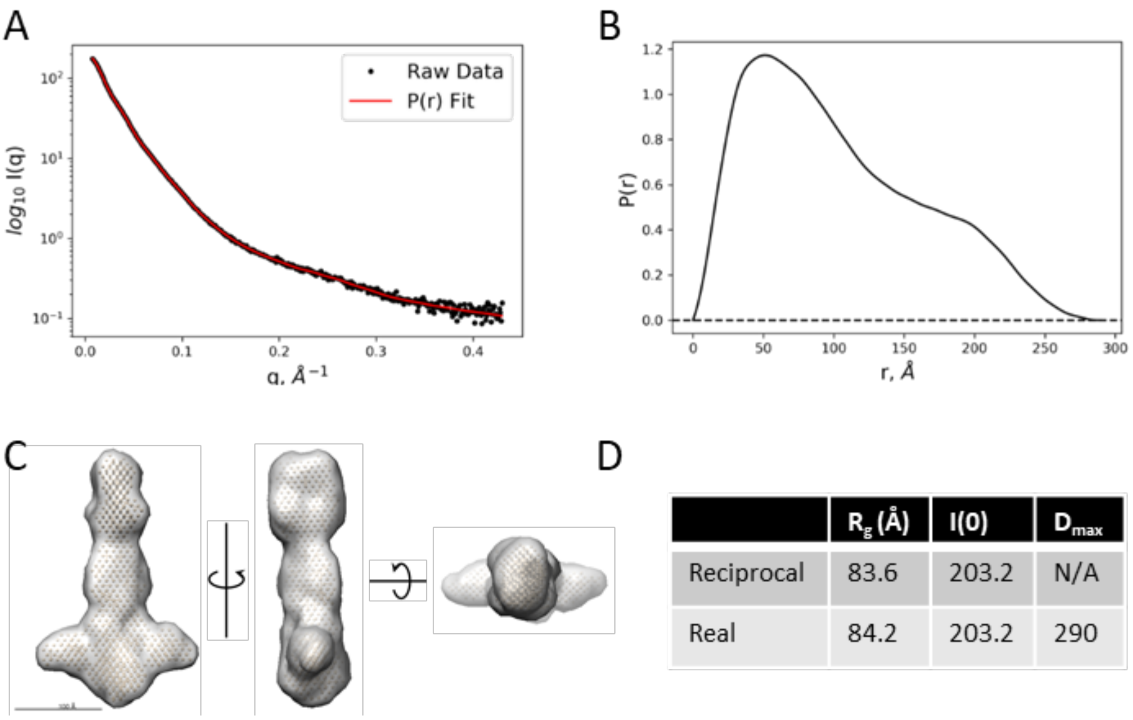

	R_g (Å)	I(0)	D_{max}
Reciprocal	83.6	203.2	N/A
Real	84.2	203.2	290

Supplementary Figure 2: SAXS data of C-terminal MUC5B.
(A) Scattering data of CT5B with the fit of the indirect Fourier Transform (IFT), from figure 1B, shown in red. (B) The IFT of the SAXS scattering data showing a maximum dimension of electron density at 309 Å and providing a real space R_g of 93.58 Å. (C) 3D reconstruction of CT5B structure showed an extended stem with double globular base. Scale bar = 100 Å. (D) Table showing structural parameters from reciprocal and real space.

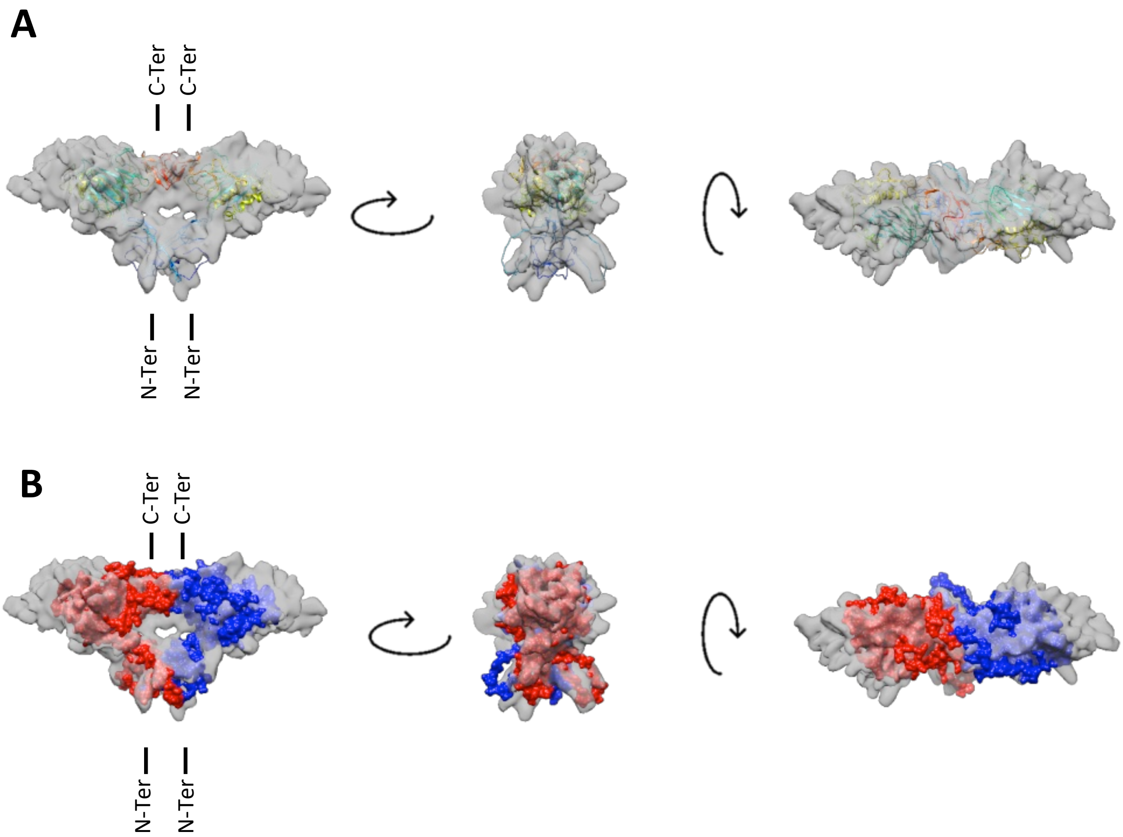

Supplementary Figure 3: Overview of CT5B base. The independently refined C2 base of CT5B with vWFD models sequentially fitted within the domains showing the location of the N- and C-termini. (A) Models with the rainbow clearly showing the N- and C-termini. (B) Ribbon representation of each domain colour coded red and blue and surface representation to also show the total occupied space.

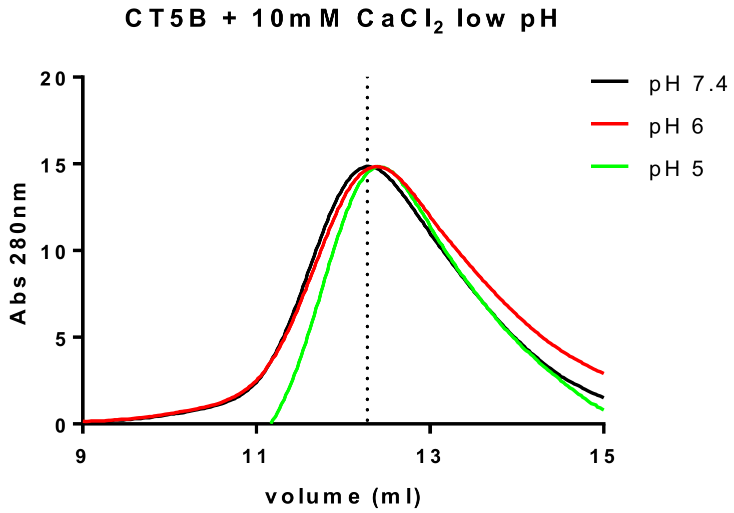

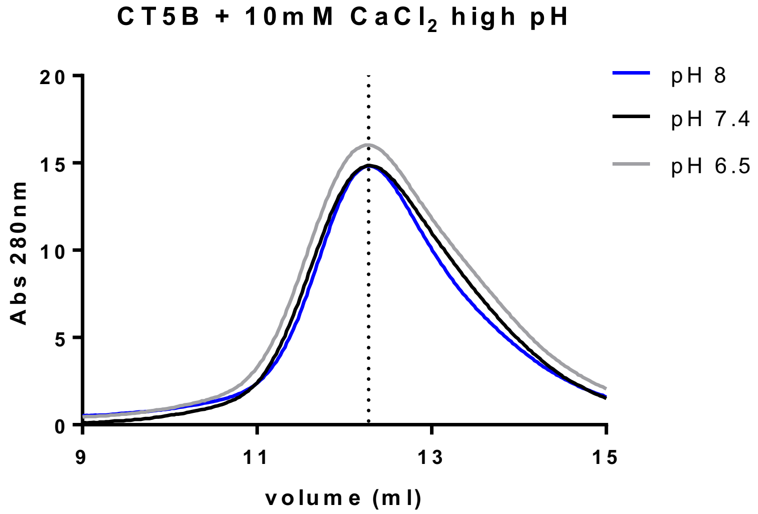

Supplementary Figure 4: Gel filtration analysis of CT5B at different pH.
CT5B was incubated in 10 mM $CaCl_2$ in different pH buffers, and then analyzed by gel filtration on a Superose 6 10/300 GL column. This experiment was performed once.

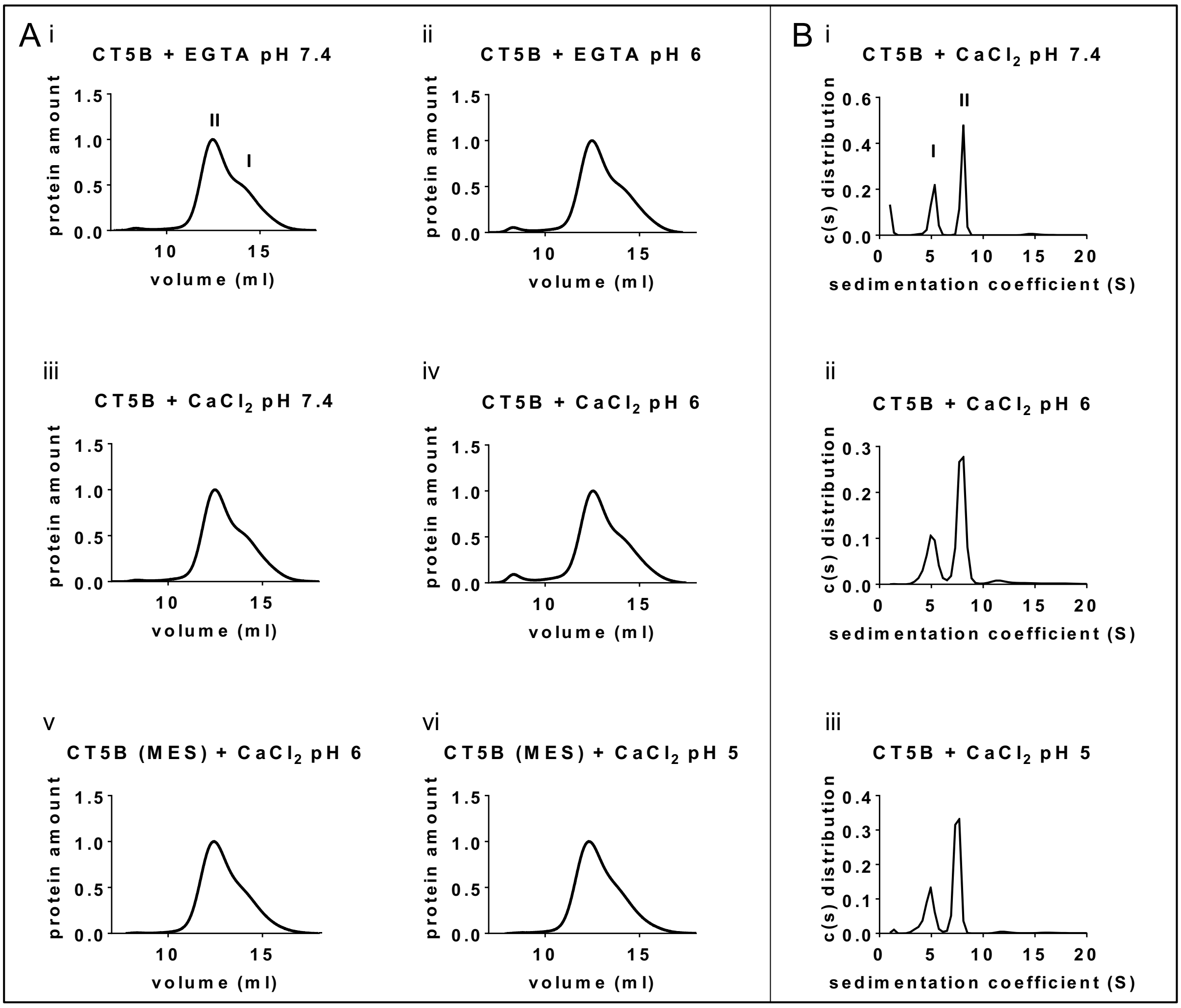

Supplementary Figure 5. Biophysical analysis of MUC5B dimerization domain in the presence and absence of calcium.
Recombinant CT5B was incubated with HBS 5 mM EGTA at pH 7.4 (Ai) and pH 6 (Aii), HBS 5 mM $CaCl_2$ at pH 7.4 (Aiii) and pH 6 (Aiv), MES 5 mM $CaCl_2$ at pH 6 (Av) and pH 5 (Avi) and analyzed by SEC-MALS. Representative graphs show the differential refractive index. CT5B showed two peaks corresponding to the dimer (peak II) and monomer (peak I). Recombinant CT5B was incubated with 5 mM $CaCl_2$ overnight at pH 7.4 (Bi), pH 6 (Bii) and pH 5 (Biii) and analyzed by AUC. CT5B showed two peaks corresponding to the dimer (peak II) and monomer (peak I). Experiments were repeated twice.
